# Supplementary material for: Maternal Fibroblast Growth Factor 21 Levels Decrease during Early Pregnancy in Normotensive Pregnant Women but Are Higher in Preeclamptic Women—A Longitudinal Study
Source: Cells. 2022 Jul 21;11(14):2251. doi: 10.3390/cells11142251 (PMC9322099; doi:10.3390/cells11142251)
Supplement: Supplementary file 1 [file cells-11-02251-s001.zip › cells-1799968-supplementary.pdf]

Supplementary Material

Maternal Fibroblast Growth Factor 21 Levels Decrease during Early Pregnancy in Normotensive Pregnant Women but Are Higher in Preeclamptic Women—A Longitudinal Study

Julieth Daniela Buell-Acosta [1], Maria Fernanda Garces [1], Arturo JoséParada-Baños [2], Edith Angel-Muller [2], Maria Carolina Paez [3], Javier Eslava-Schmalbach [4], Franklin Escobar-Cordoba [5,6], Sofia Alexandra Caminos-Cepeda [7], Ezequiel Lacunza [8], Justo P. Castaño [9,10], Rubén Nogueiras [10,11], Carlos Dieguez [10,11], Ariel Iván Ruiz-Parra [2] and Jorge Eduardo Caminos [1,*]

[1] Department of Physiology, School of Medicine, Universidad Nacional de Colombia, 11001, Bogota, Colombia; judacostabo@unal.edu.co (J.D.B.A.); mfgarcesg@unal.edu.co (M.F.G.); jecaminosp@unal.edu.co (J.E.C)

[2] Department of Obstetrics and Gynecology, School of Medicine, Universidad Nacional de Colombia,
post code: 11001, Bogota, Colombia; ajparadab@unal.edu.co (A.J.P.B.);
eangelm@unal.edu.co (E.A.M.);
airuizp@unal.edu.co (A.I.R.P.)

[3] Department of Public Health, School of Medicine, Universidad Nacional de Colombia, 11001, Bogota, Colombia; mcpaezl@unal.edu.co (M.C.P)

[4] Department of Surgery, School of Medicine, Universidad Nacional de Colombia, 11001, Bogota, Colombia; jheslavas@unal.edu.co (J.E.S.)

[5] Department of Psychiatry, School of Medicine, Universidad Nacional de Colombia, 11001, Bogota, Colombia; feescobarc@unal.edu.co (F.E.C.)

[6] Fundación Sueño Vigilia Colombiana, 111211, Bogota, Colombia

[7] School of Medicine, Universidad Pompeu Fabra, 08002, Barcelona, Spain; soficaminos2002@gmail.com (S.A.C.C.)

[8] Centro de Investigaciones Inmunológicas Básicas y Aplicadas (CINIBA), Facultad de Ciencias Médicas,
Universidad Nacional de La Plata, post code: 1900, La Plata, Argentina; ezequiellacunza@hotmail.com (E.L.)

[9] Maimonides Institute of Biomedical Research of Cordoba (IMIBIC), Reina Sofia University Hospital, 14004, Cordoba, Spain; justo@uco.es (J.P.C)

[10] CIBERObn- Physiopathology of Obesity and Nutrition, Intituto de Salud Carlos III, 28029 Madrid, Spain; ruben.nogueiras@usc.es (R.N.); carlos.dieguez@usc.es (C.D.)

[11] Department of Physiology (CIMUS), School of Medicine, Instituto de Investigaciones Sanitarias (IDIS),
Universidad de Santiago de Compostela, 15782, Santiago de Compostela, Spain

* Correspondence: jecaminosp@unal.edu.co

Table S1. Characteristic of healthy eumenorrheic women.

Variables	Healthy eumenorrheic women	P value*
Age (years)	22.3 ±3.5 (19.0 - 31.0)	
BMI (kg/m^2)	21.26 ±1.76 (18.37 – 24.45)	
SBP (mmHg)	106.95 ±9.74 (92.00- 125.00)	
DBP (mmHg)	69.00±5.93 (58.00- 79.00)	
MBP (mmHg)	81.65±6.35 (72.00- 91.33)	
Blood glucose (mg/dL)	82.20±7.47 (66.09 - 96.00)	
Insulin (μUI/mL)	9.14±5.68 (2.00 - 23.10)	
HOMA Index	1.58 ±0.87 (0.38 - 3.06)	
Total cholesterol (mg/dL)	157.40±27.22 (118.00- 208.20)	
HDL-C (mg/dL)	48.83±7.76 (38.00- 66.00)	
LDL (mg/dL)	109.52±27.29 (63.42- 182.98)	
VLDL (mg/dL)	15.20±4.65 (8.32– 25.00)	
Triglycerides (mg/dL)	85.69±19.04 (63.40– 126.00)	
C–Reactive protein	1.18 ±0.79 (0.27 - 3.16)	
Follicular Progesterone (nmol/L)	0.51±0.24 (0.17– 1.09)	0.0000
Luteal Progesterone (nmol/L)	10.76±5.52 (2.61– 20.12)	
Leptin (ng/mL) Follicular	16.52±6.63 (10.65 – 42.03)	0.0008
Leptin (ng/mL) Luteal	22.94±6.38 (12.75– 36.51)	
FGF-21 (pg/mL) Follicular	164.63 ±118.97 (38.24 - 431.29)	0.0031

FGF-21 (pg/mL)<br>Luteal	73.70±32.57<br>(25.54 -145.28)	

Abbreviations: BMI, Body mass index; HDL-C, High-Density Lipoprotein Cholesterol; LDL, Low-Density Lipoprotein; VLDL, Very Low-Density Lipoprotein; SBP, Systolic blood pressure (mmHg); DBP, Diastolic blood pressure (mmHg); MBP, Medium blood pressure (mmHg); HOMA, Homeostatic model assessment; FGF-21, Fibroblast Growth Factor 21. A p value of < 0.05 was considered as statistically significant.

Table S2. Comparison of baseline variables between preeclamptic women and healthy normotensive pregnant women during the 1st, 2nd and 3rd trimester of pregnancy.

Variables	1st trimester	2nd trimester	3rd trimester
	Healthy vs Preeclamptic P value*	Healthy vs Preeclamptic P value*	Healthy vs Preeclamptic P value*
Age (years)	-	-	-
Gestational age (weeks)	-	-	-
BMI (kg/m^2)	0.003209	0.000261	0.000000
SBP (mmHg)	0.000157	0.000024	0.000017
DBP (mmHg)	0.019085	0.006593	0.078554
MBP (mmHg)	0.001151	0.000097	0.001954
Blood glucose (mg/dL)	0.092303	0.100827	0.66653
Insulin (μUI/mL)	0.001257	0.000661	0.001301
HOMA Index	0.001180	0.000412	0.12737
Total cholesterol (mg/dL)	0.745418	0.982405	0.872114
HDL-C (mg/dL)	0.031761	0.197771	0.007896
LDL (mg/dL)	0.976787	0.880851	0.572311
VLDL (mg/dL)	0.815926	0.721656	0.941816
Triglycerides (mg/dL)	0.802943	0.728255	0.47716
C–Reactive protein	0.680187	0.000641	0.035536
Leptin (ng/mL)	0.000000	0.000000	0.000000

FGF-21	0.006462	0.016454	0.004678

The Mann–Whitney U test was used for comparisons of continuous Log transformed values. Abbreviations: BMI, Body mass index; HDL-C, High-Density Lipoprotein Cholesterol; LDL, Low-Density Lipoprotein; VLDL, Very Low-Density Lipoprotein; SBP, Systolic blood pressure (mmHg); DBP, Diastolic blood pressure (mmHg); MBP, Medium blood pressure (mmHg); HOMA, Homeostatic model assessment; FGF-21, Fibroblast Growth Factor 21. A p value of < 0.05 was considered as statistically significant.

Table S3. Comparison of serum FGF-21 levels in three gestational periods and postpartum in healthy pregnant women and healthy non - pregnant women.

			Healthy Pregnant women			
		**Luteal**	**1st trimester**	**2nd trimester**	**3rd trimester**	**Post-partum**
	**Follicular**	0.003113	0.000008	0.000343	0.675701	0.2532
	**Luteal**		0.618699	0.73812	0.000638	0.000004
Healthy Pregnant women	**1st trimester**			0.263631	5.76789E-8	1.65623E-10
Healthy Pregnant women	**2nd trimester**				0.000013	9.23714E-8
Healthy Pregnant women	**3rd Trimester**					0.055068

Comparison between serum FGF-21 levels in three gestational periods and postpartum in healthy pregnant women. Statistical differences were evaluated through the Student t-test of independent samples assuming unequal variances. Log transformed values were used for testing differences variables. A p value of < 0.05 was considered as statistically significant.

Table S4. Comparison of serum FGF-21 levels in three gestational periods between healthy pregnant and preeclamptic women.

		Women with preeclampsia		
		1st trimester	2nd trimester	3rd trimester
Healthy Pregnant women	Follicular	0.152444	0.355084	0.082853
Healthy Pregnant women	Luteal	0.0886177	0.0348624	0.000068
Healthy Pregnant women	1st trimester	0.006462		
Healthy Pregnant women	2nd trimester		0.016454	
Healthy Pregnant women	3rd trimester			0.004678

Comparison between serum FGF2-21 levels in three gestational periods in healthy pregnant women and Preeclamptic women. Statistical differences were evaluated through the Student t-test of independent samples assuming unequal variances. Log transformed values were used for testing differences variables. A p value of < 0.05 was considered as statistically significant.

Table S5. Pearson's correlation coefficient between serum FGF-21 levels and study variables in pregnant women during the 1st, 2nd and 3rd trimester of pregnancy. A p value of < 0.05 was considered as statistically significant.

Variable	Pregnant women 1st trimester		Pregnant women 2nd trimester		Pregnant women 3rd trimester	
	R-value	P-value	R-value	P-value	R-value	P-value
BMI (kg/m^2)	-0.135309	0.3293	-0.135309	0.3293	0.112535	0.4178
SBP (mmHg)	0.126336	0.3627	0.126336	0.3627	-0.099631	0.4735
DBP (mmHg)	-0.061706	0.6576	-0.061706	0.6576	-0.068658	0.6218
MBP (mmHg)	0.028594	0.8374	0.028594	0.8374	-0.086200	0.5354
Blood glucose (mg/dL)	-0.188812	0.1715	-0.188812	0.1715	0.148882	0.2826
Insulin (µUI/mL)	-0.116647	0.4009	-0.116647	0.4009	0.0402799	0.7724
HOMA Index	-0.138244	0.3188	-0.138244	0.3188	0.0596852	0.6681
Total cholesterol (mg/dL)	0.058317	0.6753	0.058317	0.6753	-0.062953	0.6511
HDL-C (mg/dL)	-0.014428	0.9175	-0.014428	0.9175	-0.095769	0.4909
LDL (mg/dL)	0.08616	0.5356	0.086164	0.5356	-0.064992	0.6406
VLDL (mg/dL)	0.133568	0.3356	0.133568	0.3356	-0.110255	0.4274
Triglycerides (mg/dL)	0.13362	0.3354	0.13362	0.3354	-0.109569	0.4303
Leptin (ng/mL)	-0.25647	0.0612	-0.25647	0.0612	-0.148248	0.2894

Abbreviations: BMI, Body mass index; HDL-C, High-Density Lipoprotein Cholesterol; LDL, Low-Density Lipoprotein; VLDL, Very Low-Density Lipoprotein; SBP, Systolic blood pressure (mmHg); DBP, Diastolic blood pressure (mmHg); MBP, Medium blood pressure (mmHg); HOMA, Homeostatic model assessment; FGF-21, Fibroblast Growth Factor 21. A p value of < 0.05 was considered as statistically significant.

Table S6. Pearson's correlation coefficient between serum FGF-21 levels and study variables in healthy eumenorrheic women during the early follicular and mid - luteal phase of the menstrual cycle. A p value of < 0.05 was considered as statistically significant.

Variable	Follicular Phase		Luteal phase	
	R - value	P-value	R - value	P-value
BMI (kg/m^2)	0.077225	0.7533	0.158931	0.5158
SBP (mmHg)	-0.001200	0.9961	-0.113731	0.6429
DBP (mmHg)	-0.107712	0.6607	-0.106645	0.6639
MBP (mmHg)	-0.067471	0.7837	-0.126497	0.6058
Blood glucose (mg/dL)	0.177049	0.4684	0.091803	0.7086
Insulin (µUI/mL)	-0.433015	0.0640	-0.052784	0.8301
HOMA Index	-0.311151	0.1947	0.034498	0.8885
Total cholesterol (mg/dL)	-0.449269	0.0536	-0.267179	0.2688
HDL-C (mg/dL)	-0.323471	0.1767	-0.432261	0.0646
LDL (mg/dL)	-0.249872	0.3022	0.030586	0.9011
VLDL (mg/dL)	0.0799702	0.7448	0.014604	0.9527
Triglycerides (mg/dL)	0.077706	0.7519	0.013082	0.9576
Leptin (ng/mL)	0.198246	0.4159	0.033259	0.8925

Abbreviations: BMI, Body mass index; HDL-C, High-Density Lipoprotein Cholesterol; LDL, Low-Density Lipoprotein; VLDL, Very Low-Density Lipoprotein; SBP, Systolic blood pressure (mmHg); DBP, Diastolic blood pressure (mmHg); MBP, Medium blood pressure (mmHg); HOMA, Homeostatic model assessment; FGF-21, Fibroblast Growth Factor 21. A p value of < 0.05 was considered as statistically significant.

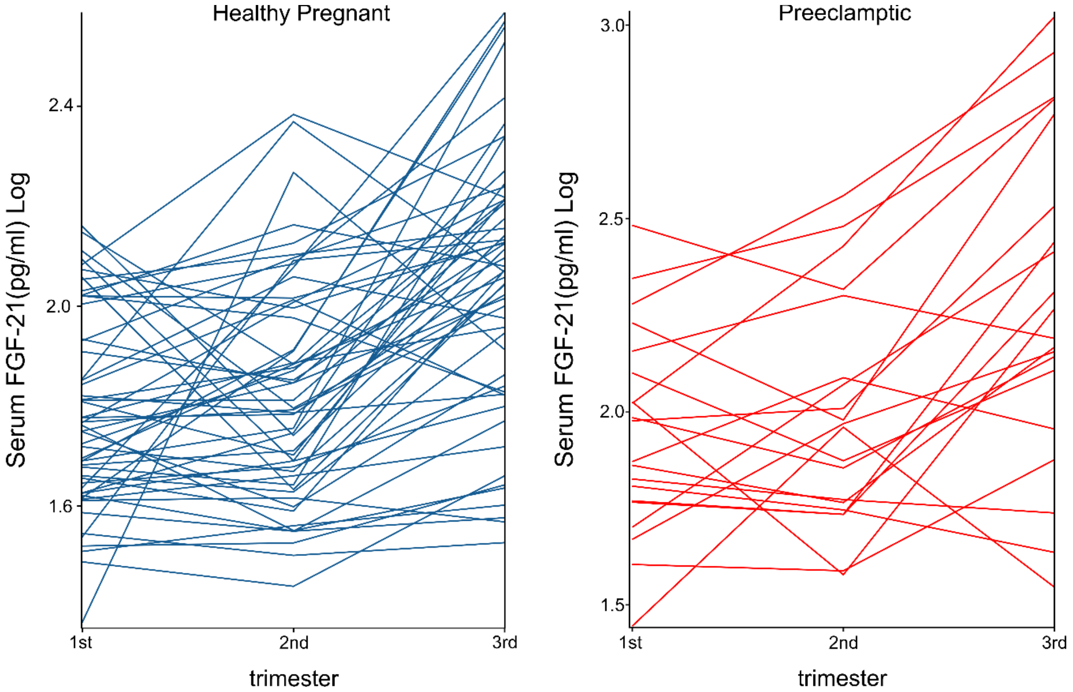

Figure S1. Diagram showing serum levels of FGF2-21 in three gestational periods in healthy pregnant women and Preeclamptic women.
